# The effect of music tempo on movement flow

**DOI:** 10.3389/fpsyg.2024.1292516

**Published:** 2024-01-29

**Authors:** Jian Zhang, Yanqun Huang, Yifan Dong, Jutao Li, Liming Zhu, Mingxuan Zhao

**Affiliations:** ^1^Key Laboratory of Mechanism Theory and Equipment Design of Ministry of Education, Tianjin University, Tianjin, China; ^2^School of Art and Design, Lanzhou Jiaotong University, Lanzhou, China; ^3^Tianjin Ren’ai College, Tianjin, China; ^4^Newton’s Grove School, Mississauga, ON, Canada

**Keywords:** music tempo, movement flow, electroencephalogram, flow experience, brisk walking

## Abstract

There has been much controversy over the effects of music tempo on movement flow. In this study, a single-factor repeated-measurement design was used to explore the effect of music tempo (fast, slow, and no music control) on movement flow by measuring both subjective experiences and objective electroencephalographic (EEG) characteristics during brisk walking. In the experiment, 20 college students walked briskly on a treadmill using EEG equipment. Each participant walked for 10 min on three different days. Their brain waves were recorded during brisk walking on a treadmill. After each walk, the participants completed a form of short flow state scale-2 (S FSS-2), which covered nine major aspects of flow. The results showed that music tempo had a significant effect on subjective experiences and objective physiological characteristics; that is, a higher subjective flow level for fast-tempo music in brisk walking and a significant enhancement of mean power values in the subconscious brain waves of the delta, theta, alpha, and beta bands for fast tempo music were observed. A fast tempo facilitated the movement flow. The findings of this study can be instructive for the use of music in exercises to improve sports training outcomes.

## 1 Introduction

Many studies have focused on exploring flow and movement flow. Flow is a harmonious and intrinsically beneficial psychological state for exercise, an immersion in a concurrent task or activity, and a sense of hitting the target in one blow, even in challenging situations (Csikszentmihalyi, 1975; Nakamura and Csikszentmihalyi, 2002). Movement flow is a flow state of heightened concentration in various sports, often associated with optimal performance (Csikszentmihalyi, 1991; Peifer et al., 2022). Issues in the study of movement flow involve the description of subjective flow experiences, causes, effects, and their interventions and measurements (Swann et al., 2012; Jackman et al., 2019, 2021). The mechanisms underlying the flow-inducing strategies during exercise are critical in the field of movement flow. Flow appears to result from the interaction between internal human states (e.g., attention, arousal, motivation, confidence, thoughts, and emotions), external factors (e.g., environmental and situational conditions), and behavioral factors (e.g., warm-up) (Swann et al., 2012). Participants’ flow intensity increases with exercise duration in sports such as Zumba, Tai Chi (Elbe et al., 2016), and other exercise games (Lee et al., 2018), indicating that continued participation in exercise has a positive effect on flow intensity and user experience. In marathon runners’ long-time exercise, flow increases during the first running hour but decreases thereafter (Wollseiffen et al., 2016). Flow usually occurs in exploratory environments with novelty, changes, and flexible outcomes or in conditions when participants pursue open-ended targets without specified targets or outcomes (Swann et al., 2019).

There are two typical types of flow measurement: subjective and objective. Jackson and Marsh (1996) and Jackson and Eklund (2002) developed a flow state scale (FSS) and dispositional flow scale (DFS), which were later simplified to the short flow state scale-2 (S FSS-2) and short dispositional flow scale-2 (S DFS-2) (Jackson et al., 2008), comprising nine items corresponding to nine dimensions of flow experience. However, researchers have reported that flow changes over time (Elbe et al., 2016; Lee et al., 2018), and a single measurement approach cannot provide trouble-free assessments of the flow experience (Jackson et al., 2008). Empirical assessments of flow are only partial reflections of experience (Jackson and Marsh, 1996; Jackson and Eklund, 2002). The flow experience might involve brain mechanisms, such as reduced activity in the dorsolateral prefrontal cortex, left inferior frontal gyrus, and amygdala (Léger et al., 2014), thereby reducing anxiety and stress (Ulrich et al., 2014, 2018; Khoshnoud et al., 2020). EEG (Electroencephalograph)--related experiments have found that the flow state is usually positively correlated with alpha and theta waves and negatively correlated with beta waves (Léger et al., 2014; Katahira et al., 2018). An increase in the delta wave power (0.5 to 4 Hz) (Rashmi and Shantala, 2022) is associated with human intoxication and immersion (Nacke et al., 2011), and delta waves become more noticeable when moving from a conscious to an unconscious state (Cacioppo et al., 2007). The mean delta power is higher in the flow state than in the non-flow state (Metin et al., 2017). Theta wave (4 to 8 Hz) (Rashmi and Shantala, 2022) is associated with unconscious meditation and concentration (Gold and Ciorciari, 2020; Khoshnoud et al., 2020), and increased activity in the theta band indicates entry into the flow state (Ulrich et al., 2014; Metin et al., 2017; Katahira et al., 2018). Alpha wave (8 to 13 Hz) (Rashmi and Shantala, 2022) activity is associated with both conscious attentional demands and unconscious relaxation, whereas higher alpha wave activity is associated with flow (Léger et al., 2014). Beta wave (13 Hz and above) (Rashmi and Shantala, 2022) is thought to be associated with conscious cognition and information processing (Ray and Cole, 1985; Moreno et al., 2020). Therefore, EEG measurements can be used as an effective objective medium to evaluate the movement flow state.

Recently, the effect of music on movement flow has become a popular research topic. Music is a type of structured sound with emotional and cognitive content that flows through time and space. People tend to achieve a flow state when immersed in music (Habe et al., 2019). Preliminary evidence indicates that music could promote flow during exercise (Pates et al., 2003; Karageorghis et al., 2008; Schuetz and Roetters, 2019). Barwood et al. (2009) and Karageorghis et al. (2012) reported measurable and relatively consistent effects of music on the behavioral and psychological states of exercisers. Laukka and Quick (2013) concluded that regularly listening to music during preparation and training could increase athletes’ positive emotions, motivation, performance, and flow. Researchers have also identified a range of benefits of music in sports, including shifting attention (association/dissociation) (Nethery, 2002), triggering or regulating specific emotions (Priest and Karageorghis, 2008; Terry et al., 2012), awakening memories and other cognitive processes (Priest and Karageorghis, 2008), controlling arousal (Ericksen, 1992), guiding flow states (Karageorghis et al., 2000), encouraging tempo movement (Karageorghis et al., 2009), increasing self-efficacy, and using mental skills (e.g., mental imagery; Garzon Mosquera and Araya Vargas, 2020). These responses to music can improve athletic performance by reducing fatigue and increasing work capacity, including exercise efficiency, endurance, and strength. The positive effects of music on exercise have attracted widespread attention. For example, Ferguson et al. (1994) investigated the effects of music emotions on karate practice performance, in which each participant performed a preselected drill following positive music, negative music, or white noise in random order and found that the enhancement of performance for positive and negative music over white noise was significant. Pain et al. (2011) found that the use of music and imagery as intervention strategies during pre-competition among soccer players had a facilitative effect on flow and perception performance. Schuetz and Roetters (2019) found significant effects of music excitation on intrinsic motivation and flow experience in equestrian sports; the experimental group (music group) had significantly higher intrinsic motivation than the control group (no music group).

Previous studies suggested that there were two speculations about music promoting movement flow: one was the enhancement of pre-exercise emotions (Terry and Karageorghis, 2011; Laukka and Quick, 2013), and the other was anxious appeasement by changing athletes’ psychological and physiological arousal as a pre-competition “stimulant” or “sedative” (Karageorghis et al., 1996). Therefore, although rhythm is an essential element of music, there is still no consensus on musical rhythms that stimulate flow during exercise. It is of interest to investigate the effect of music tempos on movement flow.

## 2 Literature review

The primary factors that influence the effects of music in sports include tempo, melody, and harmony (Karageorghis et al., 2012). Loud and positive music increases arousal, whereas soft and slow music decreases it (Copeland and Franks, 1991; Brownley et al., 1995). Music can also increase the exercise tempo, improve efficiency, and decrease relative oxygen uptake (Terry et al., 2012). The use of music during exercise can provide both psychological (dissociation and increased positive emotions) and functional (improved performance) benefits (Karageorghis and Jones, 2000; Wu et al., 2022) and contribute to promoting neuromuscular efficiency during repetitive activities of long duration (Brownley et al., 1995). Music is often used for preexercise activation (Karageorghis et al., 1996, 2010), postexercise relaxation, and rehabilitation (Terry et al., 2012). For instance, Pates et al. (2003) found that musical interventions helped control emotions and cognition. However, in high-intensity training, the effect of music on fatigue is typically negligible because the impact of internal body feedback on attention is dominant (Tenenbaum, 2001).

In recent years, considerable progress has been made in the study of the relationship between music and movement flow. Brownley et al. (1995) investigated the physiological and emotional responses of trained and untrained runners under three music conditions (no music, calming music, and fast tempo music) and found that fast or optimistic music during exercise may benefit untrained runners while being counterproductive to trained runners. Copeland and Franks (1991) studied the effects of different types of music (fast and exciting music, soft and slow music, and no music) on heart rate, fatigue perception, and time to fatigue during treadmill exercise and concluded that soft and slow music reduced physiological and psychological arousal while increasing endurance performance. Karageorghis et al. (1999) found significant correlations between music motivational characteristics (association, musicality, cultural influence, and tempo response) and flow experience in a subjective study of 334 aerobics instructors. Cultural influences and associations can be attributed to personal factors, whereas musicality and tempo responses can be attributed to musical factors. In treadmill training, musical conditions with fast, medium, and mixed tempos had higher flow intensities than the no-music control condition, whereas a paired comparison of the three tempos showed that the medium tempo had the highest flow intensity (Karageorghis et al., 2008). Karageorghis and Jones (2014) reported that there was only a weak association between the optimal choice of music tempo and a range of psychological outcomes (e.g., affective valence) at different exercise intensities. Regardless of music tempos, music reduced the number of associative thinking by 10% at all exercise intensities, and music was less popular at high intensities compared to low and medium intensities. Another study (Karageorghis et al., 2006) suggested that people prefer medium- and fast-tempo music in moderate-intensity exercises and fast-tempo music in high-intensity exercises. However, a linear relationship between exercise intensity and music tempo preference has only been partially supported in certain sports (Karageorghis et al., 2006). Exercisers usually move consciously using musical beats, and both motivational and external music at any tempo during treadmill walking can be effective in enhancing exercisers’ endurance (Karageorghis et al., 2009). Thus, the conclusions vary in different contexts and require further discussion.

In the neurophysiological and psycho-musicological sciences, the rhythmic properties of music-stimulated humans (Khalfa et al., 2008) impact their physiological arousal (Karageorghis and Jones, 2014), and many regions of the cerebral cortex are involved in music processing (Schmidt and Trainor, 2001).

Tempo is one of the most easily manipulated music attributes and is considered a key factor in musical responses (Karageorghis and Jones, 2014). Music tempo has significant effects on flow intensity during exercises (Karageorghis et al., 2008); however, there is no consensus on the effects of music tempo on movement flow. Therefore, in this study, we use music tempo as the stimulant of movement flow, controlling other musical elements, such as the concentration of association, musicality, and culture, and conducted a single-factor stimulating experiment. This study aims to investigate the effect of music tempo on movement flow during brisk walking on a treadmill, by combining subjective evaluations of flow state scales and objective EEG measurements.

## 3 Hypothesis

There is a strong correlation between the motivational qualities of music and the flow experience (Karageorghis et al., 1999). For instance, participants self-reported higher flow intensities in the fast, medium, and mixed tempo music conditions than in the no-music control condition (Karageorghis et al., 2008), and people mostly prefer fast tempo music in moderate-intensity exercises (Brownley et al., 1995; Karageorghis et al., 2006). Therefore, the following hypotheses are proposed:

*H1*: A fast tempo leads to higher subjective evaluation of movement flow during brisk treadmill walking.

*H2*: A fast tempo is more effective for unconscious brainwave stimulation during brisk walking on treadmills.

## 4 Methodology

This study was approved by the Ethics Committee of Tianjin University and the experiments were performed in accordance with the approved guidelines. A single-factor repeated-measurement design was used to investigate the effect of music tempo on movement flow during brisk treadmill walking. The experiments were conducted in a laboratory. The participants were required to visit the laboratory three times to participate in the experiments under different conditions. Each time, the participant briskly walked on the treadmill for 10 min. Each trial was scheduled simultaneously on different days to minimize the effects of fatigue and circadian rhythms. The experiments were arranged in a Latin square design to arrange the experimental sequences.

### 4.1 Variables

Music tempo (fast tempo vs. slow tempo vs. no music control) was the independent variable and the flow state scales and EEG measurements were the dependent variables. The subjective flow state was measured using a nine-item 5-point Likert scale, and objective brain waves were examined using the mean power values of EEG signals, such as delta, theta, alpha, beta, and gamma bands.

### 4.2 *A priori* power analysis

In this study, the G*Power (Faul et al., 2007) program was used to perform *a priori* power analysis at a significance level of 0.05 (*α* = 0.05), a statistical power (1-β) of 0.80, a medium effect size (*f* = 0.25; Faul et al., 2009), and a high correlation value of 0.65 between each repeated measure (Barwood et al., 2009; Karageorghis et al., 2012). Besides, the repeated measures ANOVA (analysis of variance) - within factors was used, the Nonsphericity correction was set to the default value (*ε* = 1), then the computed total sample size was 20.

### 4.3 Participants

According to the *a priori* power analysis, 21 students from Tianjin University were enrolled as participants, aged between 18 and 27 years, including eight males (mean = 21.3, SD = 3.23) and 13 females (mean = 23.5, SD = 1.15). The participants were untrained, and non-professionals (Laukka and Quick, 2013); had no regular fitness habits; had no scientific running experiences or methods of guidance; were able to use the treadmill for brisk walking; had no muscular, skeletal, respiratory, or cardiovascular system diseases; and had normal hearing.

All participants were required to meet the following requirements before each walking test: (1) At least 8 h of sleep before each experimental day and avoidance of strenuous activity to prevent cardiopulmonary or muscle function damage or abnormalities. (2) Not eating or drinking alcohol, consuming caffeine, or drinking excess water within 2 h before the experiment. Each participant signed a written informed consent form before the experiment.

### 4.4 Stimuli

The music stimuli included fast and slow tempos. Fast tempo music was strong at a beat of 150–160 bpm; whereas slow tempo music was between a narrow range, soft, soothing, and a beat of 90–100 bpm (Karageorghis et al., 2008; Wu et al., 2022). Based on previous studies (Karageorghis and Jones, 2014; Grgic, 2022), one fast and one slow tempo were selected and played repeatedly at an intensity of 75 dBA. The selected music was relatively simple, without lyrics, and had an upbeat style and obvious rhythm to stimulate positive emotions (Karageorghis and Jones, 2000; Wu et al., 2022). This simple music removed personalized preferences and was more universal; simple music helped participants improve performance in repetitive endurance exercises, music without lyrics reduced the influence of lyrical content, and obvious rhythm reduced participants’ relative oxygen uptake during the experiment (Terry et al., 2012). The upbeat style of music matched participants’ expectations (North and Hargreaves, 2008).

### 4.5 Experimental settings and equipment

**Figure 1:**
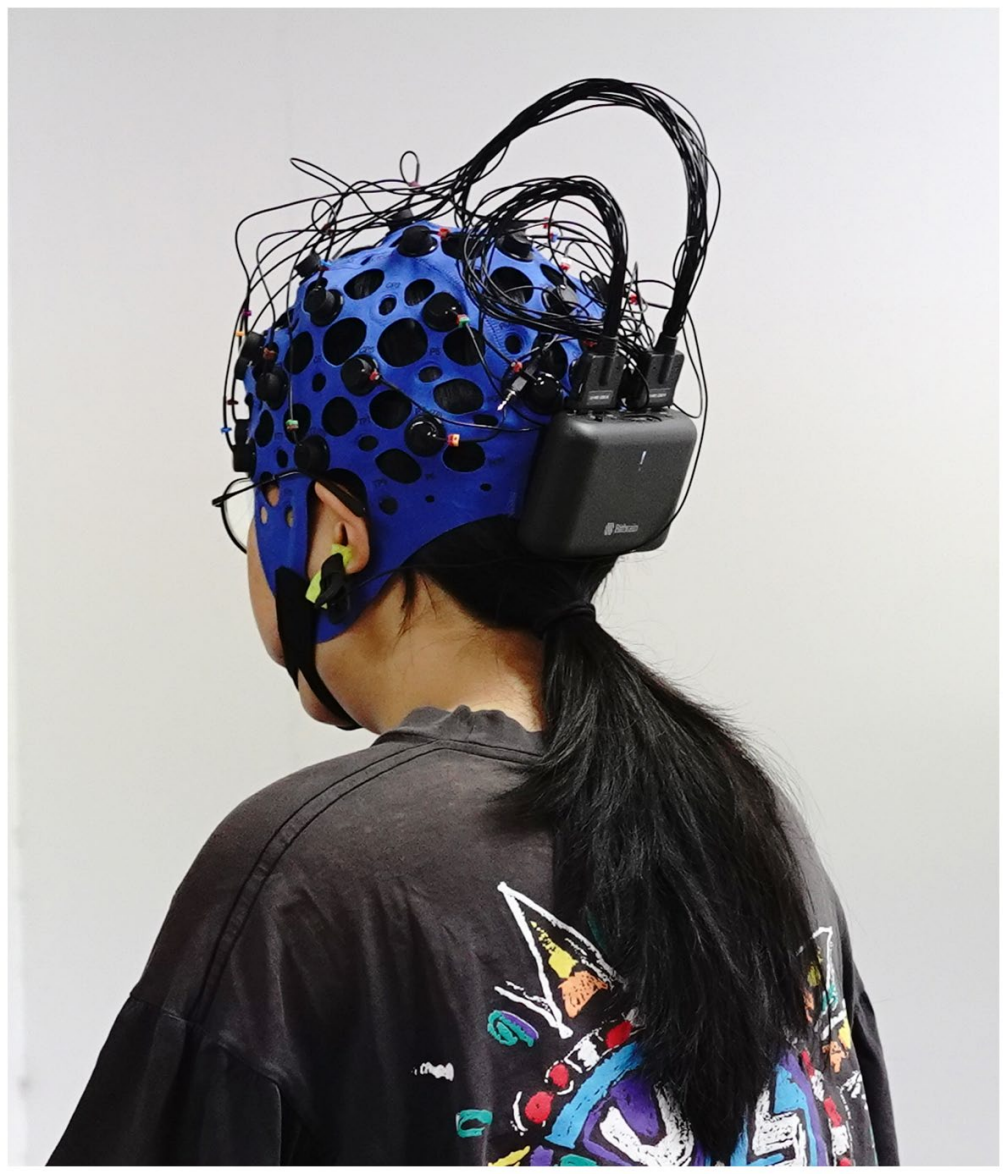
EEG equipment.

The experiments were conducted in a laboratory environment where the ambient temperature was maintained at 23 ± 2°C, and fresh air was circulated during the walking test. The treadmill was a SOLE F63 type, with a continuous motor power of 3HP, a running area of 510 × 1,550 mm, a weight of 113 kg, and a shock absorption and anti-slip system. The EEG equipment used was a 32-conductor hydroelectrode manufactured by Bitbrain (see Figure 1), including a 32-channel amplifier, electrode caps, and lead wires. A gyro was built in to resist the movement interference. There are 32 electrode points in total. The electrode positions were used in a 10–10 international standard lead system. The reference electrode (REF) was placed in the earlobe. The single-conductor sampling rate was 256 Hz and the resolution was 24 bits. Simultaneous collection of EEG data was performed using ErgoLAB 3.10.0 software during each brisk walking session. In addition, a Lenovo Rescuer-15ISK laptop was used to play music at different tempos.

### 4.6 Data collection

Based on the short FSS-2 (Jackson et al., 2008), a 5-point Likert scale was used to measure subjective flow experiences. The scale ranged from 1 to 5 (1 = “completely disagree” to 5 = “completely agree”). As listed in Table 1, nine-question items corresponding to the nine flow characteristics were included.

**Table 1:** Short flow state scale-2.

Item	Description
Challenge skill balance	I feel I am competent enough to meet the high demands of the scenario
Action awareness	I act spontaneously and automatically, without thinking
Clear goals	I know exactly what I want to do
Unambiguous feedback	I know how I am performing
Concentration on task	I am completely focused on the task at hand
Sense of control	I have a sense of complete control over what I am doing
Loss of self- consciousness	I do not care what others think about me
Transformation of time	Time seems to elapse in a different way than usual
Autotelic experience	This experience is very rewarding

Objective brain waves were examined using EEG equipment to record the mean power values of the delta, theta, alpha, beta, and gamma bands.

### 4.7 Experimental procedure

Each participant was individually tested. Before the experiment, the participants were introduced to the contents and steps of the experiment, and the use of the treadmill, correct posture for brisk walking, and requirements for completing the S-FSS scale were explained. Subsequently, each performed a two-minutes warm-up exercise without music at a speed of 5 km/h to avoid muscle injury during the experiment. A fully moistened sponge was inserted into each electrode slot of the EEG cap. After the warm-up, the participant wore the EEG cap, and a stable connection of the electrodes to the scalp was checked.

The participants walked briskly on the treadmill at a speed of 6.5 km/h (female participants) and 7 km/h (male participants; Karageorghis and Jones, 2014) for 10 min under one of the three conditions (fast tempo, slow tempo, or no-music control), with music played by speaker. To avoid the influence of visual stimuli, the participants were required to look straight ahead at a blank wall. The experimenter observed participants’ movements and ensured their safety throughout the experiment.

At the end of brisk walking, the EEG cap was removed, the participant filled out the S FSS-2 scale, and was instructed to perform appropriate stretching and recovery exercises. The entire experiment lasted for approximately 30 min.

### 4.8 Statistical analysis

First, the reliability and validity of sample data were verified using Cronbach’s α coefficient and effect size (i.e., the partial η^2^, Cohen’s d, and Adj. Cohen’s d *). The repeated one-way ANOVA was used to analyze the subjective scale data and objective EEG brainwaves data obtained in the experiment, where the independent variable was music tempo, and dependent variables were subjective scale scores and the mean power values of brainwaves (i.e., delta, theta, alpha, and beta). All statistical analyses were performed using SPSS software (IBM SPSS Statistics Subscription Trial, https://www.ibm.com/cn-zh/products/spss-statistics). We considered *p* < 0.05 to be statistically significant.

## 5 Results

### 5.1 Reliability and validity

Cronbach’s α coefficient was used for the reliability judgment, and the partial *η*^2^ effect size index was used for the validity judgment. As listed in Table 2, Cronbach’s α coefficient indicated that the internal consistency of the scales used in this experiment had a considerable degree of confidence (Jackson et al., 2008). The effect size is a measure of the experimental effect size and is unaffected by the sample size. The partial *η*^2^ (= 0.183 > 0.14) for this experiment indicated a large effect size. In most cases, Ezekiel’s formula provides a more reliable result (Wang and Thompson, 2007). Thus, according to previous research (Ivarsson et al., 2013), the effect size was adjusted in Tables 3 4 as Adj. Cohen’s d *, since the sample size was relatively small in this study.

**Table 2:** The Cronbach’s α coefficients under different rhythms.

Music Tempo	Cronbach’s α
Fast tempo	0.735
Slow tempo	0.746
No-music control	0.741

**Table 3:** The validity of flow experience.

(I) Tempo	(J) Tempo	Partial *η^2^*	Cohen’s d	Adj. Cohen’s d*
Fast tempo	No-music control	0.324	0.675	0.45
Fast tempo	Slow tempo	0.008	0.085	0.00
Slow tempo	No-music control	0.192	0.475	0.06

**Table 4:** The validity of EEG signals.

Waveband	(I) Tempo	(J) Tempo	Partial *η^2^*	Cohen’s d	Adj. Cohen’s d*
Delta	Fast tempo	No-music control	0.346	0.709	0.48
	Fast tempo	Slow tempo	0.034	0.184	0.00
	Slow tempo	No-music control	0.216	0.512	0.19
Theta	Fast tempo	No-music control	0.301	0.64	0.40
	Fast tempo	Slow tempo	0.1	0.325	0.00
	Slow tempo	No-music control	0.079	0.286	0.00
Alpha	Fast tempo	No-music control	0.272	0.596	0.34
	Fast tempo	Slow tempo	0.185	0.465	0.00
	Slow tempo	No-music control	0.006	0.078	0.00
Beta	Fast tempo	No-music control	0.295	0.63	0.39
	Fast tempo	Slow tempo	0.139	0.392	0.00
	Slow tempo	No-music control	0.025	0.157	0.00
Gamma	Fast tempo	No-music control	0.038	0.194	0.00
	Fast tempo	Slow tempo	0.0005	0.022	0.00
	Slow tempo	No-music control	0.045	0.212	0.00

### 5.2 Subjective flow experience

**Figure 2:**
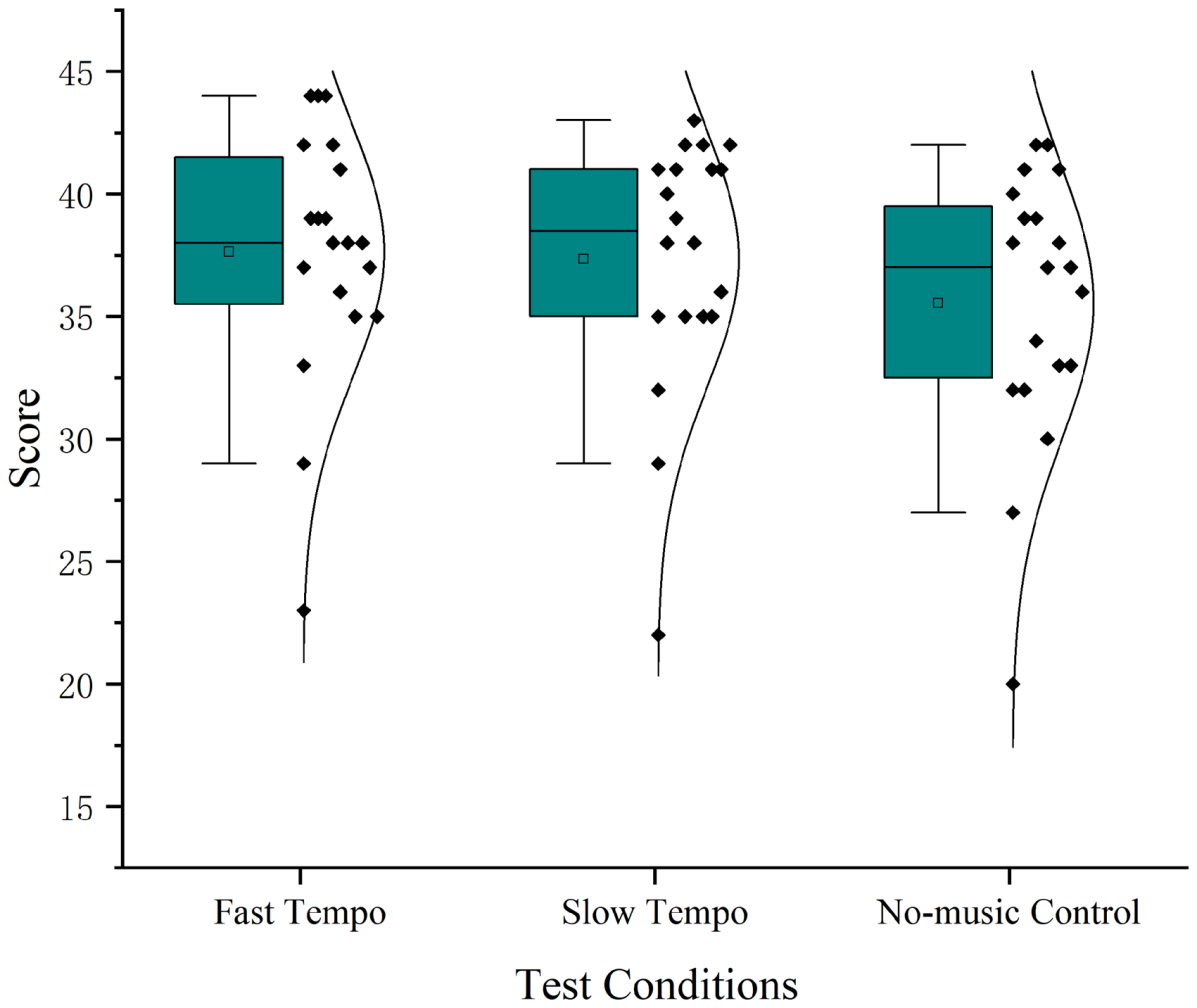
Effects of music tempos on subject flow experience.

Twenty valid responses were obtained because one participant failed to wear an EEG cap successfully. As listed in Table 5, the ANOVA results indicated that the music tempo had a significant effect on the movement flow experience score. The mean value of the movement flow score was higher for brisk walking with fast tempo, followed by slow tempo and no-music control, as shown in Figure 2. The paired comparisons with Bonferroni correction in Table 6 indicate significant differences in flow scores between no-music control and slow tempo music (*p* = 0.047), and between no-music control and fast tempo music (*p* = 0.007), with no-music control having the lowest flow experience.

**Table 5:** Tests of within-subject effects on subjective flow experience.

Source	*df*	MS	F	*p*	Partial *η^2^*
Music Tempo	2	25.8	4.243	0.022*	0.183
Error(Music Tempo)	38	6.081			

*indicates *p* < 0.05.

**Table 6:** Subjective flow experience paired comparison.

(I) Tempo	(J) Tempo	MD (I-J)	SD	*p*	Partial *η^2^*	Cohen’s d
Fast tempo	No-music control	2.100	0.695	0.007*	0.324	0.675
Fast tempo	Slow tempo	0.300	0.788	0.708	0.008	0.085
Slow tempo	No-music control	1.800	0.848	0.047*	0.192	0.475

a. *indicates *p* < 0.05; b. Multi-comparison adjustment: LSD (least significant difference).

### 5.3 Electroencephalographic signals

**Figure 3:**
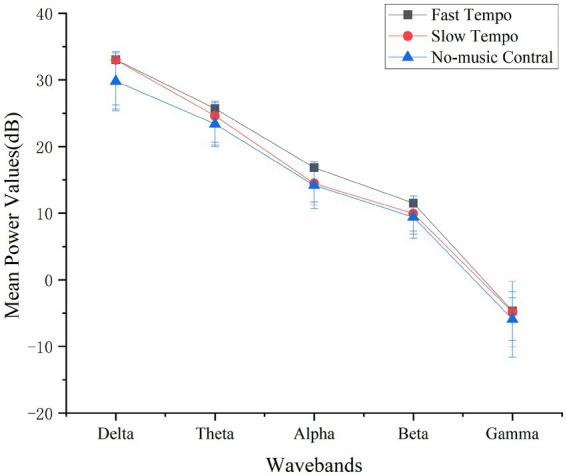
Power spectrum diagrams at different music tempos.

In the ErgoLAB software, the high-pass filter was set to 1 Hz, low-pass filter was set to 40 Hz, and band-stop filter was set to 50 Hz to exclude interference signals. The basic units of power distribution in frequency were w/Hz, mw/Hz, μw/Hz, etc., and the “dB” was usually referred to as a calculation method to facilitate the representation of large or small data and the introduction of the logarithmic law of “multiples” then the actual converted units were dBw/Hz, dBm/Hz, dBμ/Hz, etc. Subsequently, to eliminate movement artifacts, we used the EEGLAB plugin of Matlab (R2022a) and applied one of the most popular methods of artifact removal, blind source separation (BSS) (Rashmi and Shantala, 2022), to remove bad channels and bad data (Roy and Shukla, 2019). The BSS models the mixing matrix for the original and observed signals and obtains the estimated sources of artifacts. The power spectrum diagrams for different music tempos are shown in Figure 3.

**Figure 4:**
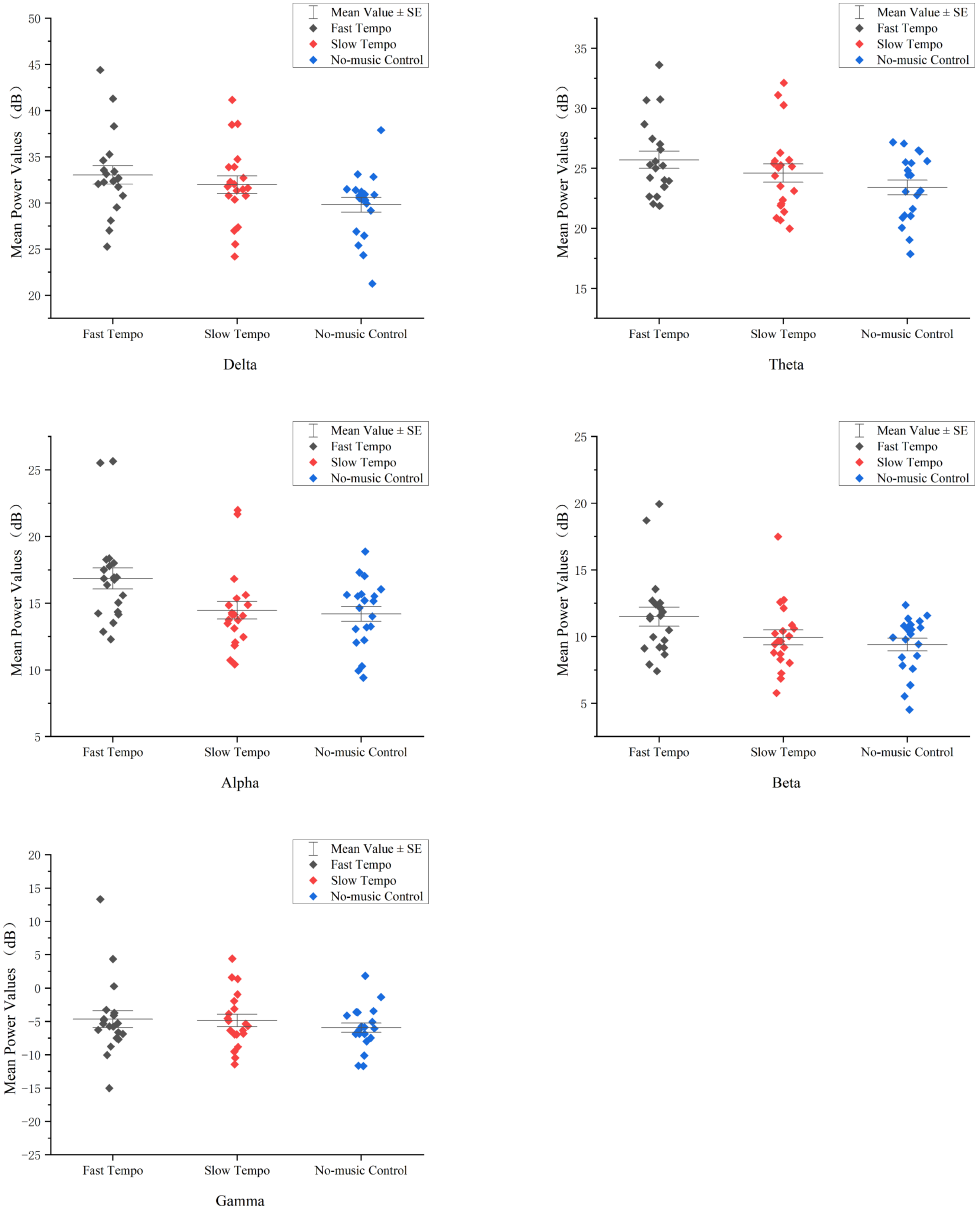
Effects of music tempos on the mean power values of wavebands.

An ANOVA was used to evaluate the effects of music tempo on the mean power values of the different wavebands, as shown in Figure 4. As listed in Table 7, The results of the repeated-measures ANOVA indicated that there were significant effects of music tempos on the mean power values of the following four bands: delta, theta, alpha, and beta, and the effect size partial *η*^2^ was large. However, the mean power value of the gamma band did not differ significantly between the three music tempos.

**Table 7:** Tests of within-subject effects on EEG signals.

Source	*df*	MS	F	*p*	Partial *η^2^*
Music Tempo (delta)	2	54.456	4.609	0.016*	0.195
Error (Music Tempo)	38	11.816			
Music Tempo (theta)	2	26.747	3.784	0.032*	0.166
Error (Music Tempo)	38	7.068			
Music Tempo (alpha)	2	42.527	4.422	0.019*	0.189
Error (Music Tempo)	38	9.617			
Music Tempo (beta)	2	23.649	3.698	0.034*	0.163
Error (Music Tempo)	38	6.395			
Music Tempo (gamma)	2	9.284	0.468	0.63	0.024
Error (Music Tempo)	38	19.857			

*Indicates *p* < 0.05.

Paired comparisons with Bonferroni correction indicated a difference in the delta waves between the no-musical control and slow tempo groups (*p* = 0.034), as listed in Table 8. There was also a significant difference in the delta waves between the no-music control and fast-tempo groups (*p* = 0.005 < 0.05). The no-music control power values were lower than those for slow and fast tempos. Fast tempo differed from the no-music control in the theta (*p* = 0.010 < 0.05), alpha (*p* = 0.015 < 0.05), and beta (*p* = 0.011 < 0.05) waves, with higher mean power values than no-music control.

**Table 8:** EEG signals paired comparison.

Waveband	(I) Tempo	(J) Tempo	MD (I-J)	SD	*p*	Partial *η^2^*	Cohen’s d
Delta	Fast tempo	No-music control	3.232	1.020	0.005^*^	0.346	0.709
	Fast tempo	Slow tempo	1.038	1.260	0.420	0.034	0.184
	Slow tempo	No-music control	2.194	0.958	0.034^*^	0.216	0.512
Theta	Fast tempo	No-music control	2.312	0.808	0.010^*^	0.301	0.64
	Fast tempo	Slow tempo	1.102	0.758	0.162	0.1	0.325
	Slow tempo	No-music control	1.211	0.945	0.216	0.079	0.286
Alpha	Fast tempo	No-music control	2.649	0.994	0.015*	0.272	0.596
	Fast tempo	Slow tempo	2.381	1.145	0.051	0.185	0.465
	Slow tempo	No-music control	0.268	0.766	0.730	0.006	0.078
Beta	Fast tempo	No-music control	2.092	0.743	0.011*	0.295	0.63
	Fast tempo	Slow tempo	1.559	0.891	0.096	0.139	0.392
	Slow tempo	No-music control	0.533	0.757	0.490	0.025	0.157
Gamma	Fast tempo	No-music control	1.252	1.441	0.396	0.038	0.194
	Fast tempo	Slow tempo	0.159	1.597	0.922	0.0005	0.022
	Slow tempo	No-music control	1.092	1.154	0.356	0.045	0.212

a. *indicates *p* < 0.05; b. Multi-comparison adjustment: LSD.

## 6 Discussion

The results indicated the significant effects of different music tempos on the flow state. Music tempo had a significant effect on the subjective flow experience, with a higher flow level for fast and slow tempo music during brisk walking, indicating that H1 was partially supported. The subjective scale measurement showed that both fast and slow tempos helped participants achieve movement flow, and the difference in subjective feedback between the two tempos on movement flow was not significant. The objective EEG measurement showed that music tempo had a significant effect on objective EEG signals, with fast tempo music resulting in significant enhancement of the mean power values in the delta, theta, alpha, and beta bands, indicating that H2 was verified.

Fast and slow tempos resulted in a high subjective evaluation of movement flow during brisk treadmill walking, suggesting that tempo did not influence the stimulation of movement flow. These results support previous findings that music can improve exercise efficiency (Terry et al., 2012), and that music intervention helps control emotions and cognition (Pates et al., 2003). However, it was not the case that music tempo is one of the main factors affecting the effectiveness of sports (Karageorghis et al., 2012): soft and slow music could decrease arousal (Copeland and Franks, 1991; Brownley et al., 1995), and medium-to-fast tempo music is preferred during low-to-moderate intensity exercise (Karageorghis et al., 2006). This could be because the subjective evaluation is a *post hoc* evaluation after the end moment, which could be affected by external factors, such as music melody and content (Karageorghis et al., 2012), while the flow changes over time (Elbe et al., 2016; Lee et al., 2018). Thus, the subjective evaluation of the experience after the experiment revealed a certain degree of uncertainty.

The results of EEG data analysis revealed a significant effect of music tempo on EEG signals. The powers of the delta, theta, alpha, and beta bands increased in response to the fast tempo music. Experimental results support the involvement of several areas of the cerebral cortex in music processing (Schmidt and Trainor, 2001). The increase in delta wave power proved that fast and slow tempo music made people more engaged in exercise, which was consistent with the fact that an increase in delta wave power is associated with human intoxication and immersion (Nacke et al., 2011). The mean delta power was higher in the flow state than in the non-flow state (Metin et al., 2017). The increase in theta wave activity suggests that fast-tempo music is more conducive to immersion. Theta waves are associated with concentration (Gold and Ciorciari, 2020; Khoshnoud et al., 2020), and increasing the activity of theta waves in the participants during the experiments indicated entering a flow state (Ulrich et al., 2014; Metin et al., 2017; Katahira et al., 2018). Higher alpha wave activity reflects a more relaxed and calmer state with fast tempo music, because alpha wave activities are associated with attentional demands. Higher alpha wave activity is associated with flow, and higher alpha wave activity was observed during the experiment, indicating entering a flow state (Léger et al., 2014). An increase in beta wave activity indicates that fast-tempo music enhances cognitive engagement, supporting the notion that beta waves are associated with cognition and information processing (Ray and Cole, 1985; Moreno et al., 2020). The results indicated that fast tempo music was more effective for unconscious brainwave stimulation and that fast tempo music was more easily stimulated movement flow.

In summary, there were disagreements between the subjective and objective evaluations: the subjective evaluation focused more on experiences after testing, whereas the objective EEG measurement recorded the duration of the entire period, which was more about the experience of the entire process. Therefore, for the entire process, we concluded that fast tempo music was more conducive to stimulating movement flow than slow tempo music or no music control. This finding supports the idea that listening to fast and optimistic music during exercise may be beneficial for untrained runners (Brownley et al., 1995). These results are inconsistent with the findings that soft and slow music reduced physiological and psychological arousal and increased endurance performance (Copeland and Franks, 1991). This might have been caused by the different exercise intensities of the exercisers and music tempos that stimulated movement flow under various exercise intensity conditions.

## 7 Conclusion

In this study, we combined an experimental method of flow state scale measurement with a physiological method that uses EEG signals to test the effect of music tempos on the experience of movement flow. The results showed that fast tempo music could generate better movement flow and more effectively stimulate an unconscious state of flow. At the application level, these results can serve as a useful guide for future exercises or training, particularly for single repetitive movements. In the early stages of exercise and training, in case of repetition of a single movement, fast tempo music can be used to stimulate the flow experience of the exerciser and effectively improve the exercise and training effect. In the middle and late stages of exercise and training, in case of familiarity with the essentials of movement, soft and slow tempo music can be used depending on the needs of specific sports and training programs. At the theoretical level, the application of the objective measurement of EEG on flow could be promising for detecting the entire dataset of the experimental process.

However, this study has some limitations. The occurrence of movement flow is elusive and unpredictable and flow measurements remain highly difficult. Understanding the causal mechanisms of the flow experience is usually descriptive, and the factors influencing flow have not been clearly, thoroughly, or comprehensively elucidated. In addition, the flow state was closely related to the specific task settings in the experiment, and more evidence is required to extend this to other activities. Furthermore, owing to the complexity of music, the current study did not consider the individual emotional effects of music on movement flow, such as participants’ favorite self-selected rhythms and lyrics with emotional elements. In future studies, the effects of other elements, such as musical complexity, mixed tempo conditions, participants’ music preferences, music intensity adaptation, music tempo, and gender differences, on movement flow should be further studied.

## Data availability statement

The original contributions presented in the study are included in the article/Supplementary material, further inquiries can be directed to the corresponding author.

## Ethics statement

The studies involving humans were approved by The Ethics Committee of Tianjin University. The studies were conducted in accordance with the local legislation and institutional requirements. The participants provided their written informed consent to participate in this study.

## Author contributions

JZ: Conceptualization, Data curation, Formal analysis, Methodology, Project administration, Software, Visualization, Writing – original draft, Writing – review & editing. YH: Funding acquisition, Resources, Supervision, Validation, Writing – review & editing, Writing – original draft. YD: Conceptualization, Data curation, Methodology, Visualization, Writing – original draft. JL: Supervision, Validation, Writing – review & editing. LZ: Supervision, Writing – review & editing. MZ: Validation, Writing – review & editing.
