# Supplementary material for: The effect of music tempo on movement flow
Source: Front Psychol. 2024 Jan 29;15:1292516. doi: 10.3389/fpsyg.2024.1292516 (PMC10860678; doi:10.3389/fpsyg.2024.1292516)
Supplement: Supplementary file 1 [file Table_1.DOCX]

Supplementary Material

# Supplementary Data

You can find supplementary data at the following links.

DOI：10.6084/m9.figshare.24031833
